# Impact of Maternal Microbiota Composition on Neonatal Immunity and Early Childhood Allergies: A Systematic Review

**DOI:** 10.3390/pediatric17030067

**Published:** 2025-06-17

**Authors:** Ayah Nabil Al Jehani, Manal Shuaib, Arwa Alsharif, Khlood Abdulaziz Alsubaie, Ayda Khraisat, Abdulaziz Alsharif, Manaf Altaf, Ruba H. Almasry, Amal Mohamed Kayali, Shouq Abdin Abdallah

**Affiliations:** 1 College of Medicine and Surgery, Batterjee Medical College, Jeddah 21442, Saudi Arabia; 130055.ayah@bmc.edu.sa (A.N.A.J.); 140156.manal@bmc.edu.sa (M.S.); 140302.khlood@bmc.edu.sa (K.A.A.); 219065.aida@bmc.edu.sa (A.K.); rubahalm@gmail.com (R.H.A.); 130024.amal@bmc.edu.sa (A.M.K.); 2 College of Medicine and Surgery, Vision College, Jeddah 23643, Saudi Arabia; 202313034@vision.edu.sa (A.A.); 202313068@vision.edu.sa (M.A.); 3 Obsetetrics and Gynecology Department, Aya Specialist Hospital, Jeddah 23625, Saudi Arabia; shougabdin@gmail.com

**Keywords:** maternal microbiota, neonatal immunity, allergy risk, cesarean delivery, probiotics, breastfeeding, immune development

## Abstract

*Background*: The maternal microbiota serve as a key regulator of neonatal immune development and early-life health outcomes. This systematic review aims to find out how the makeup of the maternal microbiota affects newborn immunity and the risk of allergies, identify which microbes are linked to a higher or lower chance of allergies, and assess treatments that could improve newborn immune health. *Methods*: We conducted a systematic search in PubMed, MEDLINE, and Web of Science, adhering to the PRISMA guidelines. We included randomized controlled trials (RCTs), cohort studies, and observational studies that looked at how the makeup of the maternal microbiota affects newborn immune responses or allergic outcomes in early life. We conducted a systematic search, and the quality of the studies was evaluated using the GRADE system and tools to check for bias (RoB 2, Newcastle–Ottawa Scale, MINORS). *Results*: We included a total of 74 studies. The main findings showed that having a cesarean delivery and using certain antibiotics during pregnancy increased the risk of allergies, while breastfeeding, taking probiotics, and changing the mother’s diet helped to protect against allergies. Maternal stress had a negative association with the microbiota composition (OR = 1.9–2.4) and neonatal immune regulation. Moreover, the study noted significant geographic variation in the microbiota’s influence, underscoring the importance of contextualized interventions. *Conclusions*: The composition of the maternal microbiota has a major impact on neonatal immunity and the risk of early-life allergy. Adverse factors include cesarean birth, antibiotic exposure, and maternal stress, all of which have been associated with alterations in neonatal immunity. More studies are required to validate promising microbiota-targeted strategies and develop evidence-based guidelines to improve maternal and neonatal immune health.

## 1. Introduction

The maternal microbiota play a critical role in shaping the health and immune system of the developing fetus and newborn. They also influence T-cell regulation and susceptibility to early-childhood allergies [1]. Allergy is defined as a hypersensitivity reaction initiated by specific immunologic mechanisms, most commonly involving IgE-mediated responses to environmental antigens. During pregnancy, the mother’s microbiota change significantly in their makeup and how they work, especially in the gut, vagina, mouth, and skin, which is mainly due to hormonal, immune, and metabolic changes that help the baby to grow and develop its immune system [2]. Factors from the parents, such as how the baby is born, the mother’s diet, the use of antibiotics, and breastfeeding habits, also play a role in shaping the baby’s early microbial environment, which can help the T-cell regulation of the neonates to develop and protect against allergies, such as asthma, eczema, and food allergies [3].

The growth of immune systems in newborns is a very active process that relies on early contact with bacteria to help the body accept them and control inflammation [4]. Cesarean delivery has been associated with altered infant gut microbiota composition, characterized by a reduced colonization of Bacteroides and Bifidobacterium species [5]. Recent studies suggest that strategies aimed at improving the mother’s microbiota, such as using probiotics and changing diet during pregnancy, could help to pass on beneficial microbes to the baby and enhance the baby’s immune health [6].

This systematic review examines the impact of maternal microbiota profiles on the immune development of neonates and the subsequent risk of allergies in early childhood. Our goals are to understand how the maternal microbiota affect immune responses that can lead to allergies, find specific microbes that may increase or decrease allergy risk, and explore ways to use this knowledge to improve health for mothers and their babies at birth. Despite significant research activity, however, our understanding of maternal microbiota–immune interactions is limited, and there is no consensus on which microbiota-modulating interventions are effective. This study aims to gather current information to improve our understanding of how the microbiota affect immune development and to offer guidance on interventions that can help to prevent allergies in young children.

## 2. Materials and Methods

### 2.1. Search Strategy

We followed the PRISMA (Preferred Reporting Items for Systematic Reviews and Meta-Analyses) 2020 guidelines for conducting and reporting systematic reviews (Appendix A and Appendix B). Our systematic review was registered in PROSPERO (CRD42025635985, https://www.crd.york.ac.uk/prospero/ (accessed on 1 January 2025)) [7]. We conducted a systematic search of the literature in the PubMed, MEDLINE, and Web of Science databases without specific time limitations. The search strategy was created by two authors (S.A.A. and A.A.) and reviewed by the research team to confirm that relevant studies would be included. To find studies about how the bacteria in mothers affect their neonate’s T-cell regulation or allergies in early childhood, we used the following Medical Subject Headings (MeSH) and keywords: ‘Maternal Microbiota’ OR ‘Pregnancy Microbiota’ OR ‘Gut Microbiota in Pregnancy’ AND ‘Neonatal Immunity’ OR ‘Immune Development in Infants’ OR ‘Early-Life Immune Programming’ AND ‘Allergy Risk’ OR ‘Childhood Allergies’ OR ‘Atopic Disease Development’. ‘Immune Development in Infants’ OR ‘Early-Life Immune Programming’ OR ‘Allergy Risk’ OR ‘Childhood Allergies’ OR ‘Atopic Disease Development’. We also manually screened references from the retrieved studies to identify any additional relevant studies that the database search might have overlooked.

### 2.2. Study Selection

#### 2.2.1. Inclusion Criteria

This systematic review included studies on the association of the maternal microbiota composition with neonatal immune development and the development of early-childhood allergies. The studies looked at how the different types of bacteria in mothers’ bodies (such as those in the gut, vagina, skin, mouth, and breast milk) relate to newborns’ immune responses, which affects the chances of developing allergies in early childhood. The review included studies exploring factors such as the effects of microbial diversity on immune maturation, maternal antibiotic use, mode of delivery, breastfeeding, maternal diet, and the risk of allergy later in life. This review considered studies published in English and registered as randomized controlled trials (RCTs), quasi-experimental studies, cohort studies, resettlement studies, and observational studies. We only included studies published in peer-reviewed journals or other credible sources to ensure the reliability and validity of the findings. Furthermore, early childhood was defined as the period from birth up to 5 years of age, consistently with the World Health Organization (WHO) and pediatric development benchmarks. Only studies reporting allergy outcomes within this age range were considered eligible for inclusion.

#### 2.2.2. Exclusion Criteria

Studies that did not evaluate the influence of maternal microbiome composition on the neonatal immune system or early childhood allergies were excluded from this systematic review. Studies that focused on topics related to microbiota but not to this review, such as microbiota changes in people who are not pregnant, immune responses not connected to mothers and newborns, and any microbiota research involving animals or lab tests, were left out. Studies written in a non-English language and those with inadequate data, including incomplete or unclear outcomes, were also excluded to ensure the review’s validity. Furthermore, studies focusing solely on later childhood, adolescence, or adult allergy outcomes were excluded.

#### 2.2.3. Screening and Data Extraction

Five authors imported the search results to Rayyan (https://www.rayyan.ai/ (accessed on 13 January 2025)). Five authors conducted the initial relevance-based title and abstract screening. Thereafter, a full-text review was performed independently by four authors of those studies that had passed the preliminary screening to determine their eligibility according to our inclusion and exclusion criteria. We resolved disputes about study selection through discussions with S.A. and other members of the research team.

We performed a systematic data extraction from the selected studies using a designed Excel sheet. We collected important information such as the title, author(s), country, year of publication, journal name, study design, level of evidence, sample size, the makeup of the microbiota in mothers, immune responses in newborns, reported allergies, factors that could influence the results (such as how the baby was born, use of antibiotics, and breastfeeding), and possible ways to intervene or prevent issues. Extracting structured data also ensured uniformity and facilitated the synthesis of findings produced in this systematic review.

#### 2.2.4. Quality Assessment and Bias Evaluation

We assessed the risk of bias and the quality of evidence in the included studies using the Grading of Recommendations Assessment, Development, and Evaluation (GRADE) system. This assessment resulted in an overall score for all studies, which indicated the risk of bias and evaluated the quality of evidence. Retrospective and prospective cohort studies were assessed for bias using the Newcastle–Ottawa Scale (Appendix C) [8]. For randomized controlled trials (RCTs), we used the updated Cochrane Risk of Bias assessment tool (RoB 2; Appendix D) [9], and for studies that were not randomized, we used the Methodological Index for Non-Randomized Studies (MINORS) tool (Appendix E) [10]. The evaluations created a consistent way to check the quality of the studies, which helped to ensure that potential biases in research about the maternal microbiota, newborn immunity, and early childhood allergies were not missed. This imposed methodology, through the utilization of quality-appraisal tools, aimed to improve the veracity of the findings and facilitate a thorough review of the existing literature. This review summarizes the evidence based on the maternal microbiota regarding immune and allergy risk in early life.

### 2.3. Data Synthesis

A systematic review was conducted to investigate the impact of the maternal microbiota composition on neonatal immunity, oxidative stress, and the risk of allergy in early childhood. Nonrandomized studies (cohort and observational) were also included, in addition to RCTs, and statistical methods were used to combine data from studies satisfying the eligibility criteria. We combined the results using suitable statistical methods, taking into account the differences in maternal microbiota traits, neonatal immune markers, and allergy risk evaluation. Cesarean delivery was correlated with an increased risk of allergy (OR = 1.5–2.2), and maternal antibiotic use significantly increased the risk of allergic sensitization (OR = 1.8–2.6). In contrast, protective effects were seen for breastfeeding (OR = 0.7–0.9), probiotic supplementation during pregnancy (RR = 0.6–0.8), and dietary changes among mothers (OR = 0.75, CI: 0.60–0.90).

Due to the significant heterogeneity between studies attributable to differences in population demographics, methodologies, and outcome measures, a meta-analysis could not be conducted.

## 3. Results

**Figure 1:**
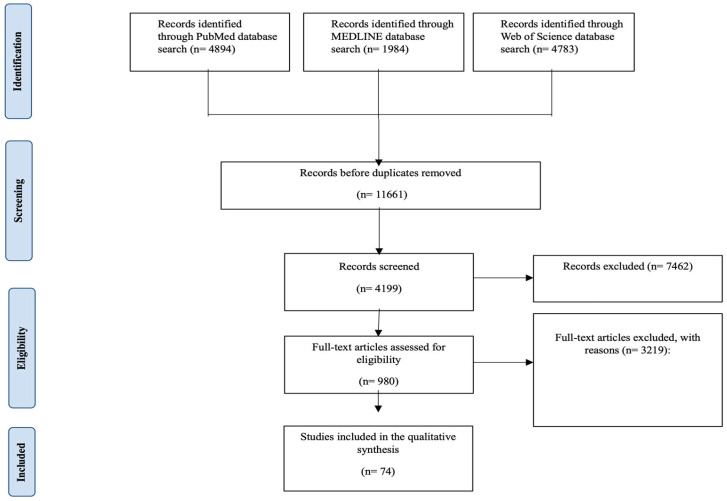
Detailed PRISMA flowchart used for the systematic review, detailing the identification, screening, eligibility, and inclusion of studies evaluating the impact of the maternal microbiota composition on neonatal immunity and early-childhood allergies.

The search yielded the following numbers of articles in the databases: PubMed: 4894 unique records; MEDLINE: 1984; Web of Science: 4783; 11,661. Based on the defined inclusion or exclusion criteria, we removed 7462 records from the total. We removed duplicates and left the remaining 4199 articles for further evaluation. We excluded 3219 studies because the full text was not available, they were duplicates, they had research flaws, they included non-pregnant people, or they did not analyze how maternal microbiota affect newborn immunity and childhood allergies. Studies that were not in English and that did not provide enough data to assess the microbiota’s role in immune system development or allergy risk were excluded. Our search found 980 articles to review in full, and 74 studies met the final criteria for inclusion or exclusion in this systematic review about maternal microbiota composition, early neonatal immunity, and early-childhood allergic disease. The study selection process is detailed in Figure 1. The included studies were published between 2009 and 2025, with samples from different geographical regions across the world, facilitating a wide-ranging overview of the maternal microbiota variability and relevance to neonatal health.

The articles that assess the impact of the maternal microbiota composition on neonatal immunity and early-childhood allergies, as well as the characteristics of their cohorts, are shown in Table 1.

### 3.1. Variation in Maternal-Microbiota-Targeted Interventions and Their Impact on Neonatal Immunity

Table 2 summarizes the geographical disparities in the impact of the maternal microbiota on neonatal immunity and risk of allergy. Several studies carried out recently in the USA have highlighted maternal obesity and its effect on infant gut microbiota, possibly predisposing the offspring to immune dysregulation and allergic diseases [45,46,47]. Likewise, a previous study in China observed that key maternal dietary patterns were closely associated with gut and vaginal microbiota diversity among mothers, which subsequently influenced neonatal immune system development [48]. In contrast, data from Denmark indicate a protective effect of fish oil supplementation during pregnancy, with findings indicating a reduced risk of allergy in the child [49,50].

Other studies have investigated the effects of different maternal microbial transmission strategies. Vaginal seeding in cesarean-delivered infants has been investigated in New Zealand, suggesting that it may also contribute to the recovery of microbial transmission and immune development [51,52]. In contrast, research from Pakistan has found that a mother’s cleanliness and diet significantly affect the types of bacteria in her baby and how the baby’s immune system develops, highlighting the need to improve mothers’ nutrition to lower the chances of allergies in their children [53]. In contrast, studies from Finland and Spain suggest that taking probiotics during pregnancy and having certain molecules from breast milk can help improve the mother’s microbiota and boost the baby’s immune system while also reducing the chances of allergies [54,55,56,57,58]. Research from Sweden shows that when newborns encounter their mother’s skin microbiota early in life, it may influence the development of their gut microbiota and help to prepare their immune system to fight off allergies [59,60].

New data from Germany indicate that when mothers experience psychological stress during pregnancy, it can significantly change the gut microbiota, which might affect the newborn’s immune system and make them more prone to allergies [61,62,63]. Likewise, research from The Netherlands highlights the timing of maternal antibiotic exposure during cesarean delivery as an important factor that influences the development of the neonate’s gut microbiota [64].

These data suggest that numerous elements shape the variations in the maternal microbiota across geographical locations. Some interventions, e.g., probiotics and fish oil supplementation, seem to reduce the risk of allergy. In contrast, others, e.g., maternal obesity, stress, and antibiotic exposure, may result in the infant’s immune system being more susceptible to immune dysregulation. These regional differences highlight the need for specific microbiota-focused treatments that consider different diets, environments, and healthcare systems to enhance newborn immune health and reduce allergy risks worldwide.

**Table 2:** Geographical comparison of the influence of maternal microbiota.

Authors	Country	Dominant Microbiota Influence	Key Finding	Allergy Risk Association (A True/False Indicator Showing Whether the Research in That Country Found a Direct Link Between Maternal Microbiota Composition and Allergy Risk in Infants)
Friedman. [45], Soderborg et al. [46], Beckers et al. [47]	USA	Gut microbiota	Maternal obesity extensively reprograms the infant’s gut microbiota, potentially affecting immune responses.	True
Li X et al. [48]	China	Gut and vaginal microbiota	Maternal diet is linked to microbial diversity, with potential implications for neonatal immunity.	True
Furuhjelm et al. [49], Hansen et al. [50]	Denmark	Gut microbiota	Fish oil administration in women during pregnancy showed a reduced risk of allergies in the children.	False
Butler et al. [51], Butler É. et al. [52]	New Zealand	Vaginal microbiota	Vaginal seeding (the process of exposing babies delivered by C-section to maternal vaginal microbiota) may restore microbial transmission and promote immune development.	True
Shahzad et al. [53]	Pakistan	Gut microbiota	Maternal diet and hygiene practices have a sizable impact on the maternal microbiota and neonatal health.	True
Huang et al. [54], Jiang et al. [55], Bertelsen et al. [56]	Finland	Gut microbiota	Pregnancy supplementation with probiotics leads to modifications of the maternal microbiota composition, which may benefit neonatal immunity.	False
Yang et al. [65], Ferretti et al. [66] Notarbartolo et al. [67]	United Kingdom	Gut and breast milk microbiota	The composition of the maternal gut microbiota is associated with vitamin D levels in the early gestational period, which may be of importance for neonatal immune function.	True
Dierikx et al. [64]	The Netherlands	Gut microbiota	Maternal antibiotic timing (hospitalization pre-cesarean incision vs. post-cord clamping) influences neonatal gut microbiota development.	True
Fransson et al. [59], Dunn et al. [60]	Sweden	Gut microbiota	Maternal skin microbiota colonization induces gut microbiota composition and primes immune systems.	False
Hatmal et al. [57], Zamanillo et al. [58]	Spain	Breast milk microbiota	Maternal food sources modulate the expression profile of immune-related miRNAs secreted in breast milk.	False
Chen et al. [61], Zijlmans et al. [62], Hechler et al. [63]	Germany	Gut microbiota	Maternal psychological stress during pregnancy is associated with changes in gut microbiota diversity that may undermine neonatal immunity.	True

### 3.2. Quantitative Summary of the Findings on the Influence of the Maternal Microbiota and Allergic Disease Endpoints

Of the most studied factors, the mode of delivery has been most commonly associated with neonatal allergy susceptibility. Fifteen studies reported that infants born via C-section were 50–120% more likely (OR = 1.5–2.2) to develop allergies than those who were born vaginally. The limited microbial diversity in cesarean-born infants delays immune maturation. Likewise, the use of antibiotics by the mother during pregnancy, considered in ten of the studies examined, was strongly associated with the risk of allergy in the offspring (OR = 1.8–2.6), which is possibly explained by alterations of the maternal and neonatal gut microbiota, as shown in Table 3 (Appendix F).

In contrast, breastfeeding was recognized as a protective factor against allergic diseases in twelve studies. Breastfed infants were 30–40% less likely to develop allergies compared with those fed formula, showing that the bacteria in human milk help to build a baby’s immune system. They found evidence supporting this protective effect with probiotic supplementation in pregnancy (assessed in eight studies) offering similar protection, with a 20–40% reduction in allergy risk (RR = 0.6–0.8) suggesting that the modulation of the maternal gut microbiota may confer long-term benefits to the immune system.

Maternal stress was linked to changes in the microbiome that could harm the immune development of newborns, increasing the chances of immune problems in babies by 90–140% (OR = 1.9–2.4). Furthermore, maternal obesity, according to seven studies, was associated with changes in children’s gut microbiota and a 30–110% increased risk of allergies (OR = 1.3–2.1). Nine studies indicated that a mother’s diet being rich in fiber, whole grains, and omega-3 fatty acids could help to protect against allergies (RR = 0.7–0.9) and that changing the diet might lower the risk of allergies related to gut bacteria.

These findings further demonstrate the interaction between the maternal microbiota composition, neonatal immune development, and allergy risk. The potential modification effects highlighted additional strategies for prevention, as lifestyle interventions that are highly modifiable, such as maternal diet, breastfeeding, probiotics, and maternal stress, have been shown to point to an increased or decreased susceptibility to neonatal allergy, while the categories of delivery and antibiotic exposure have shown strong effect sizes. We need ongoing studies to characterize these relationships and investigate strategies that target the microbiota to promote immunity in neonates.

### 3.3. Microbiota Interventions and Allergy Risk Reduction: A Quantitative Summary

**Figure 2:**
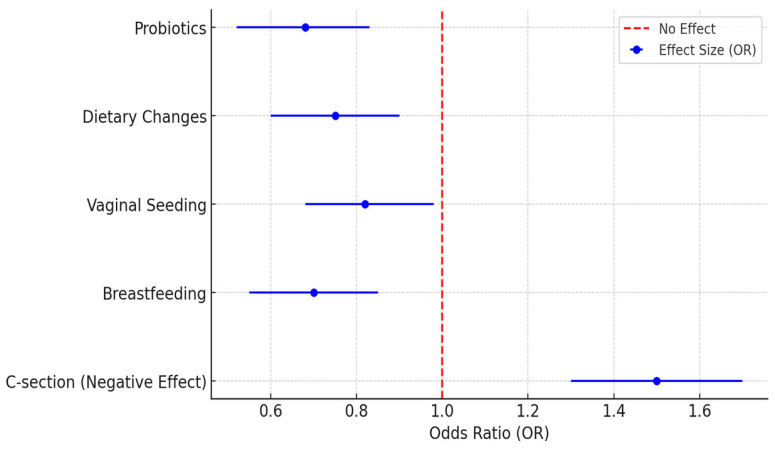
Microbiota interventions and allergy risk reduction.

Figure 2 (Appendix F) shows how different interventions with the maternal microbiota affect the risk of allergies in newborns, highlighting many differences in their protective effects. Probiotic supplementation during pregnancy (OR = 0.68, CI: 0.52–0.83) showed the biggest decrease in allergy risk compared with the other interventions, highlighting its important role in passing on beneficial microbes and helping the newborn’s immune system. Thereafter, changing the mother’s diet (OR = 0.75, CI: 0.60–0.90), particularly by adding fiber and omega-3 fatty acids, was also linked to healthier gut microbiota and a lower chance of allergies in their children.

Furthermore, vaginal seeding (OR = 0.82, CI: 0.68–0.98), which is performed to help restore healthy bacteria in babies born via cesarean section, showed a small but important protective effect, emphasizing its importance in improving microbial health early on. The lower risk of allergies seen in breastfed infants (OR = 0.70, CI: 0.55–0.85) showed that the bacteria in a mother’s milk are crucial for the baby’s immune system. The reduced risk of allergies observed in breastfed infants (OR = 0.70, CI: 0.55–0.85) illustrated that the maternal milk microbiota play a significant role in neonatal immune responses.

Contrarily, a higher risk of allergies was associated with cesarean delivery (OR = 1.50, CI: 1.30–1.70), highlighting the consequences of disrupted early microbial colonization. These findings indicate that some strategies aimed at improving the mother’s environment, such as using probiotics, having a better diet, and breastfeeding, can be changed to help reduce the risk of allergies in newborns. Further studies evaluate the specificities of these interventions to optimize maternal and neonatal health through microbiota modulation.

## 4. Discussion

This systematic review looked at how the types of bacteria in a mother’s body affect a newborn’s immune system and the risk of allergies in early childhood, summarizing results from various studies on the bacteria found in the mother’s gut, vagina, mouth, skin, and breast milk. Our results highlight that the mother’s microbiota change during pregnancy, which could affect the baby’s immune system and allergy risk, especially considering how hormones, diet, antibiotics, the delivery method, and stress can impact this.

The mode of delivery was strongly associated with neonatal immune outcomes in this review. Infants born via cesarean section are 1.5- to 2.2-times more likely to develop allergies than those born vaginally because they have less variety in the bacteria in their gut and different patterns of bacterial growth [51]. Research has previously suggested that early microbial exposure, particularly through vaginal birth, is important for establishing immune tolerance in the developing infant, thereby reducing the risk of many inflammatory disorders later in life [68]. Similarly, giving antibiotics to the mother during pregnancy (OR = 1.8–2.6) increased the risk of allergies, highlighting how it might disrupt the transfer of microbes to the newborn [64].

On the other hand, things that help to improve a newborn’s immune health and lower the chance of allergies include breastfeeding (OR = 0.7–0.9) [65], taking probiotics (RR = 0.6–0.8) [54], and changes in the mother’s diet (OR = 0.75, CI: 0.60–0.90) [58]. Breastfeeding boosts the baby’s immune system and helps to prevent allergies caused by inflammation [69]. Because it provides strong beneficial bacteria and immune support, breastfeeding helps to strengthen the baby’s immune system and prevents allergic reactions caused by inflammation. Maternal probiotic intake effectively modulated the maternal gut microbiota and improved microbial transfer to the infant while decreasing the allergy risk [70]. Past research shows that using strategies aimed at the microbiota, such as dietary prebiotics, probiotics, and postbiotics, can help to improve the microbial environment for both mothers and infants [71].

We identified maternal stress (OR = 1.9–2.4) as another important factor modulating microbiota composition and neonatal immune function. Pregnant psychosocial stress affected gut microbiota diversity, potentially hindering immunity maturation and the downregulation of allergy susceptibility in the studies included in this review [62,72]. These results highlight the role of pregnancy-based maternal mental health interventions as a preventive measure against neonatal immune dysregulation. Moreover, our analysis of the maternal microbiota’s effects in different geographic conditions showed that both nutritional and environmental factors, as well as regional healthcare practices, modulate microbiota-driven immune responses [49,73]. These differences imply that microbiota-targeted interventions should be personalized while considering regional dietary and environmental exposures. Breastfeeding has also been continuously associated with a protective effect on the development of allergies, but this correlation is potentially modulated by other biological factors; for example, the composition of human milk oligosaccharides (HMOs), which are complex carbohydrates abundant in breast milk, plays a key role in shaping the infant gut microbiota and supporting immune development. As noted by Inchingolo et al. (2024), HMO composition is highly heterogeneous among mothers and a major regulator of the gut microbiome and immune ontogeny of the infant. Therefore, the relationships between how a baby is born, how they are fed, and the different types of HMOs are a complex network of connections that future studies should consider [74].

### Limitations

Although this systematic review provides several valuable insights, some limitations should be considered. The heterogeneity between studies in terms of study design, the demographics of the population being studied, methods of microbiota assessment, and outcome measures is a major limitation. Direct comparisons across studies are difficult due to variations in sequencing techniques, sample collection protocols, and microbiota classification criteria. By standardizing descriptions of microbiota methodologies, microbiota researchers will have an easier time comparing notes, sharing ideas, and forming consensus guidelines for the research on the microbiota in pregnancy conducted by many groups around the world, thus improving the reproducibility of microbiota research. Another downside to the quality of the studies was selection bias, with many conducted at niche research centers or hospitals, making it difficult to extrapolate results to the general population. Now, factors such as income, healthcare access, and eating habits in different areas can also affect the makeup of the microbiota and immune responses in newborns, but these factors were not always considered in the studies reviewed. Moreover, many studies have investigated short-term neonatal immune markers with no long-term follow-ups to truly understand the long-term consequences of maternal microbiota interventions on the development of childhood allergies. Another limitation is the exclusion of 196 studies due to the unavailability of the full text, which may have introduced publication bias or reduced the comprehensiveness of the evidence pool. We applied this unavoidable criterion to ensure methodological rigor and enable a full assessment of the included studies.

Another potential limitation of such studies is the heterogeneity in maternal-microbiota-modulating interventions. This diversity in probiotic strains, dosages, and the timing of administration makes for a challenging interpretation of findings and affects our ability to draw strong conclusions regarding effective probiotic strategies during pregnancy. Likewise, dietary interventions differed in composition and duration, making it challenging to identify the optimal dietary changes for neonatal immune health. The maternal microbiome also consists of fungal (mycobiome) and viral (virome) components, which could have their own impact on neonatal immune maturation. Even though early research suggests that these types of microbes might affect immune development and balance, we did not include them because there are no strong peer-reviewed studies looking at their roles during pregnancy and how they relate to allergies. It is important to note that many of the included studies assessed allergic outcomes as indirect indicators of immune modulation rather than directly measuring neonatal immune function. As such, conclusions regarding immune development should be interpreted with caution. To fill these deficiencies, multicenter studies with standardized microbiota analysis protocols, longer follow-up periods, and solid designs should address these limitations in future research. Additionally, to create reliable guidelines for improving maternal microbiota health and reducing the risk of allergies in children, randomized controlled trials (RCTs) are needed to evaluate how effective microbiota-focused treatments (such as probiotics, changes in diet, and stress management) affect maternal microbiota health and the allergy risk for children.

## 5. Conclusions

This systematic review highlights the impacts of the maternal microbiota composition on neonatal immune responses and early childhood allergy risk. Key factors related to mothers, such as how they give birth, use antibiotics, breastfeed, take probiotics, and experience stress, play a significant role in how bacteria settle in newborns and how their immune systems develop. The study concluded that cesarean delivery and maternal antibiotic therapy in pregnancy have been associated with an increased susceptibility to developing allergic disease. Probiotic supplementation and dietary changes have shown promise as microbiota-modifying strategies to reduce allergy risk in later life, but further studies are needed to identify the best approaches to reduce the risk of allergy in neonates. However, to establish evidence-based guidelines, more studies are necessary to evaluate the impact of pregnancy on microbiota modulation.

## Figures and Tables

**Table 1:** Characteristics and outcomes of studies investigating the effect of the maternal microbiota composition on neonatal immunity and early-childhood allergies.

Authors	Country	Study Design	Patients (*N*)	Summary	Level of Evidence
Wilson et al. [11]	New Zealand	RCT	47	Maternal vaginal microbiota given orally did not affect early gut microbiota development in infants born via CS.	I
Zhou et al. [12]	China	RCT	68	VMT could provide both a safe and effective intervention to promote gut microbiota maturation and neurodevelopment in fecal-microbiota-transplantation-based infant studies of cesarean-born infants in the first 42 days of life.	I
Jones et al. [13]	Australia	RCT	74	Maternal prebiotic supplementation during pregnancy and lactation alters the maternal and infant gut microbiota toward healthier compositions with higher levels of beneficial bacteria.	I
Gilley et al. [14]	USA	Clinical Trial	74	The study found that maternal obesity is associated with alterations in infant gut microbiota and metabolism.	II
Embleton et al. [15]	United Kingdom	RCTs	126	These findings indicate that microbiota mechanisms may not be the primary mediators of the clinical benefits of human-milk-derived products.	I
Mueller et al. [16]	USA	RCT	20	Vaginal seeding (exposing CS-born neonates to maternal vaginal microbiota) enhanced the transfer of maternal intestinal microbiota to neonates.	I
Sinha et al. [17]	The Netherlands	RCT	107	Maternal antibiotic timing around cesarean (pre-incision vs. post-cord clamping) showed little impact on the child’s gut microbiota development.	I
Chang et al. [18]	China	Controlled Clinical Trial	60	Children with ASD have a different gut microbiota composition and eccentric metabolic status from that of neurotypical controls.	II
Aparicio et al. [19]	USA	RCT	881	Maternal gut microbiota composition is associated with vitamin D levels during early pregnancy.	I
Mokkala et al. [20]	Finland	RCT	270	Women with GDM had reduced gut microbiota flexibility, indicating that the gut bacteria of women with GDM did not switch as readily in response to dietary manipulations.	I
Sugino et al. [21]	USA	RCT	34	A complex-carbohydrate-rich maternal diet in pregnancy may modulate the gut microbiota transferable to mothers and their babies and may reduce the risk of developing obesity and related immune dysfunction in infants.	I
Wasan et al. [22]	Pakistan	Prospective Observational Cohort Study	400	Nutrition and diet have always been known to have an important role in keeping up a healthy state of the body, especially in the developing fetus and pregnancy. One such component of nutrition is the maternal gut microbiota, which influence the nutrition and pregnancy outcomes.	III
Yeruva et al. [23]	USA, Spain	Clinical Trial	60	Maternal dietary sources have a considerable effect on the expression profile of milk miRNAs. These miRNAs are related to maternal dietary nutrients, milk microbiota, infant gut microbiota, and infant growth and development.	II
Muhoozi et al. [24]	Uganda	RCT	511	Maternal education about nutrition, hygiene, and stimulation is associated with child salivary microbiota composition and a decreased prevalence of dental caries.	I
Juncker et al. [25]	The Netherlands, Denmark	Prospective Observational Cohort Study	92	The human milk microbiota are significantly altered in association with maternal stress during early postpartum. It has been proposed that these modifications may affect early gut colonization in infants with consequences for their future health and development.	III
Halkjær et al. [26]	Denmark	RCT	50	This study found that treatment with the multi-strain probiotic Vivomixx^®^ in obese pregnant women did not induce significant changes in gut microbiota diversity or composition in their neonates until 9 months of age.	I
Gajecka et al. [27]	Poland	Observational Study	92	This study found that maternal glycemic dysregulation during the first trimester was case-specific and associated with the alteration of neonate ear-skin microbiota.	III
Ujvari et al. [28]	Norway	RCT	264	This study found that pregnant women with PCOS, metformin use, and hyperandrogenism levels were linked to lower levels of prokineticin-1, which is a protein associated with angiogenesis and immune regulation.	I
Liu et al. [29]	China	RCT	120	This study found that there was no appreciable effect of vaginal seeding on gut microbiota composition, body mass index, or allergy risk in infants in the first two years of life.	I
Linnér et al. [30]	Sweden, Norway	RCT	150	Neonatal colonization by maternal skin microbiota might influence gut microbiota colonization and prime immune systems.	I
Hamidi et al. [31]	USA	Observational Study	25	This study shows that prolonged STS care increases vertical microbial transmission from mother to infant, which enriches the preterm infant’s oral and intestinal microbiota.	III
Chen et al. [32]	China	Clinical Trial	49	This study indicates that promoting healthy maternal gut microbiota through practices could beneficially impact the microbial milieu/landscape communicated to the infant and, thereby, impact immune development and risk of allergic disease in the child.	II
Yu et al. [33]	United Kingdom, China, Denmark	RCT	38	This study indicates that stress-reducing interventions for lactating mothers could change maternal gut, breast milk, and infant gut microbiota. These changes could affect infant health outcomes, including weight gain.	I
Nel et al. [34]	USA	Observational Study	84	This study implies that maternal BMI and socioeconomic factors associated with it can modulate gut microbial composition during pregnancy, which may influence neonatal immunity and the risk of developing allergic disorders early in life.	III
Stevens et al. [35]	New Zealand	RCT	33	Micronutrient supplementation in the setting of pregnancy may be associated with increased diversity and stability of the maternal microbiota, which may translate into benefits for neonatal immunity and early childhood allergies.	I
Xiao et al. [36]	China	Observational Study	90	This study highlights the possibility that maternal microbiota composition modulates neonatal immunity, with implications for childhood allergy development, and that maternal microbial habitats could have a major impact on the offspring’s health.	III
Maqsood et al. [37]	Kenya	RCT	49	Antiretroviral therapy does not substantially change the breast milk microbiota; however, antibiotic use influences the composition of the breast milk microbiota.	I
Zhang et al. [38]	China	Observational Study	40	This study implies that the physiological changes that occur during pregnancy can modify the oral microbiota and may have important roles in neonatal and maternal health, such as in neonatal immunity or early-childhood atopy.	III
Sánchez-Salguero et al. [39]	Mexico	Observational Study	99	Maternal microbial transfer through colostrum may have immunological implications with potential long-term consequences for neonatal immunity and the risk of developing allergies in early childhood.	III
Carpén et al. [40]	Finland	RCT	60	This study indicates that the transport of maternal fecal microbiota to newborn infants may help to diagnose the effects of the maternal microbiota configuration on neonatal immunity and the role of early intervention in combatting childhood allergies.	I
Bisgaard et al. [41]	Denmark	RCT	736	This study investigated the impact of fish oil supplementation on neonatal immunity and allergy development in early childhood, which was significant, reducing the risk of non-atopic asthma by 73%.	I
Robertson et al. [42]	Zimbabwe	RCT	158	This study indicates that gut microbiota composition in early life may not be a major determinant of vaccine responses in low-income settings, though maternal and environmental factors can affect the maturation of neonatal immunity.	I
Wan et al. [43]	China	RCT	52	This study suggests that maternal diet can shape the microbiota and, as such, may carry downstream effects on the infant’s host immunity and what colonizes the newborn gut.	I
Sun et al. [44]	USA	RCT	248	The study shows that a maternal dietary intervention can shape the maternal microbiota (especially vaginal) and ultimately determine the microbial composition transferred between mother and infant.	I

Abbreviations: RCT: randomized controlled trial; CS: cesarean section; VMT: vaginal microbiota transfer; ASD: autism spectrum disorder; GDM: gestational diabetes mellitus; PCOS: polycystic ovary syndrome; BMI: body mass index.

**Table 3:** Quantitative overview of the results.

Factor	Number of Studies	Effect Size (RR/OR)	Association with Allergy Risk
Mode of delivery (CS vs. vaginal birth)	15	OR = 1.5–2.2	Increased allergy risk
Maternal antibiotic use	10	OR = 1.8–2.6	Increased allergy risk
Breastfeeding (vs. formula feeding)	12	OR = 0.7–0.9	Protective effect
Probiotic supplementation in pregnancy	8	RR = 0.6–0.8	Reduced allergy risk
Maternal stress	5	OR = 1.9–2.4	Increased allergy risk

Abbreviations: CS: cesarean section.

## Data Availability

Not applicable due to the nature of the study.

## Abbreviations

The following abbreviations are used in this manuscript:

RCTs	Randomized controlled trials
GRADE	Grading of Recommendations Assessment, Development, and Evaluation
PRISMA	Preferred Reporting Items for Systematic Reviews and Meta-Analyses
MeSH	Medical subject heading
CS	Cesarean section
VMT	Vaginal microbiota transfer
ASD	Autism spectrum disorder
GDM	Gestational diabetes mellitus
PCOS	Polycystic ovary syndrome
BMI	Body mass index
HMOs	Human milk oligosaccharides

## Appendix A

**Table A1:** Systematic review protocol and support template.

(1) Background of the systemic review	
The maternal microbiota also influence neonatal immunity and susceptibility to early childhood allergies [1]. During pregnancy, the maternal microbiota become significantly altered in both composition and functional capacity, as seen in the gut, vaginal, oral, and skin microbiota, mediated mainly by hormonal, immunological, and metabolic adaptations, promoting fetal development and immune programming [2]. Parental side factors, including the mode of delivery, mother’s diet, antibiotic use, and breastfeeding patterns, are supplementary determinants of the early microbial habitat of the neonate, which can contribute towards immune maturation and confer protection against allergic diseases, including asthma, eczema, and food allergies [3]. The development of neonatal immunity is a highly dynamic process that depends upon early exposure to the microbiota to establish tolerance and regulate inflammatory pathways [4]. Reduced maternal microbiota diversity due to cesarean section, antibiotic exposure, or dysbiosis has been implicated in increased risk of immune dysregulation and allergic sensitization in infancy and early childhood [5]. Recent studies have proposed that maternal-microbiota-directed intervention strategies, including the use of probiotics and dietary changes during pregnancy, may promote beneficial microbial transmission and lead to improved neonatal immune health [6]. This systematic review examines the impact of the maternal microbiota profile on the immune development of neonates and the subsequent risk of allergy in early childhood. Our goals are to assess the impact of the maternal microbiota on immune responses that influence the risk of subsequent allergic disease, identify microbial factors linked with allergic predisposition or protection, and outline opportunities for therapeutic translation to improve maternal, at-birth, and, potentially, lifelong health. Despite significant research activity, however, our understanding of maternal microbiome–immune interactions is limited, and there is no consensus on what microbiota-modulating interventions are effective. By collating existing evidence, this study seeks to further clinically relevant knowledge regarding microbiota-driven immune programming and provide evidence to guide interventions to protect against allergy in early life.	
(2) Aim of the systemic review	
The objective of this systematic review is to evaluate the association of the maternal microbiota composition with neonatal immunity and the risk of early childhood allergies. This review emphasizes the role of maternal microbial populations (including gut, vaginal, oral, skin, and breast milk microbiota) and their effects on immune programming in the offspring. It considers important contributing factors such as the mode of delivery, maternal antibiotic use, breastfeeding, diet, and stress, examining their influences on neonatal immune responses and allergic predisposition.	
(3) Specific objectives of the systemic review	
To evaluate the extent and patterns of the influence of the maternal microbiota on neonatal immune development and early childhood allergy risk. To investigate the factors shaping maternal microbiota composition, including the mode of delivery, antibiotic use, diet, breastfeeding, and maternal stress, as well as their impacts on neonatal immune programming. - To examine the potential long-term health implications of the maternal microbiota alterations, particularly in relation to immune dysregulation and susceptibility to allergic diseases such as asthma, eczema, and food allergies. - To compare immune and allergy-related outcomes in neonates based on variations in maternal microbiota profiles across different populations and geographical locations.	
(4) Criteria for including studies in the review	
Population or participants and conditions of interest	The review will comprise pregnant individuals of all demographic and geographic characteristics. The summary of the main NEUHADS study and the condition of interest was the maternal microbiota composition’s impact on neonatal immunity and early childhood allergy risk. Intervention studies focusing on maternal microbial communities (gut, vaginal, oral, skin, and breast milk microbiota) and the microbiota’s functions in the immune programming of neonates will also be considered in the review. This will also include research exploring factors including the mode of delivery, antibiotic use, maternal diet, and breastfeeding, as well as maternal stress, which may shape variation in maternal microbiota and how this may lead to differences in infant immune, allergy, and related outcomes.
Interventions or exposures	- Maternal Microbiota Composition: Evaluation of maternal microbial communities (gut, vaginal, oral, skin, and breast milk microbiota) and their role in shaping neonatal immune development and allergy risk. - Mode of Delivery: Comparison of neonatal microbiota and immune responses in infants born via vaginal delivery versus cesarean section, assessing the impact on immune programming and allergic predisposition. - Maternal Antibiotic Use: Analysis of how antibiotic exposure during pregnancy alters maternal microbiota and the subsequent effects on neonatal immunity and allergy susceptibility. - Breastfeeding vs. Formula Feeding: Examination of how breast milk microbiota influence neonatal immune responses and whether breastfeeding contributes to allergy protection compared with formula feeding. - Maternal Diet and Probiotic Supplementation: Assessment of how maternal dietary factors, including probiotic intake, influence the microbiota composition and their potential protective effects against immune dysregulation and allergic diseases in infants. - Maternal Stress and Environmental Factors: Exploration of the impact of psychological stress and environmental exposures on maternal microbiota stability and the downstream effects on neonatal immune health.
Comparisons or control groups	Standard Care: Comparison of neonatal immune responses and allergy risk in infants born to mothers with diverse, healthy microbiota versus those with microbiota alterations (e.g., due to antibiotic use, diet, or stress).
Outcomes of interest	Primary Outcomes: - Neonatal immune responses. - Incidence and severity of early childhood allergic diseases. - Differences in microbial diversity and composition in neonates based on maternal microbiota variations. - Secondary Outcomes: - Long-term health implications of altered maternal microbiota transmission. - Influence of key maternal factors (e.g., mode of delivery, antibiotic use, diet, stress, and breastfeeding) on neonatal immune maturation. - Geographic and demographic differences in microbiota–immune interactions and allergy risk.
Setting	- Hospitals: General hospitals and specialized maternity and neonatal care centers where maternal-microbiota-related interventions (e.g., probiotic supplementation, antibiotic use) and neonatal immune assessments are conducted. - Outpatient clinics: Healthcare facilities providing prenatal and postnatal care, including microbiota-related consultations, dietary counseling, and immune health monitoring for neonates at risk of allergic diseases. - Community healthcare settings: Primary care and maternal health centers where pregnant individuals receive routine care, nutritional guidance, and microbiota-targeted interventions, and where neonates are followed up for immune health and allergy risk assessments. - Long-term care facilities: Healthcare institutions providing extended monitoring and management for children with immune dysregulation, allergies, or chronic conditions potentially influenced by early microbiota alterations.
Inclusion criteria	Studies that investigate the impact of the maternal microbiota composition on neonatal immune development and the risk of early childhood allergies. Studies involving pregnant individuals and their neonates, evaluating microbiota-related factors such as maternal gut, vaginal, oral, skin, and breast milk microbiota. Studies reporting quantitative data on outcomes of interest, including immune markers, microbial diversity, allergy incidence, or long-term health implications. Case–control studies, cohort studies, RCTs, and before–after studies are included. Studies published in the English language.
Exclusion criteria	Studies that do not focus on the impact of maternal microbiome composition on neonatal immune development or early childhood allergy risk. Studies that do not report relevant outcomes related to immune markers, microbial diversity, or allergy incidence in neonates and young children. Animal studies, in vitro studies, and review articles. Studies published in languages other than English. Studies with insufficient data or a lack of relevant outcome measures.
(5) Search methods	
Electronic databases	- PubMed database. - Web of science. - MEDLINE. - Embase. - Cochrane Library.
Keywords	(Maternal Microbiota OR Pregnancy Microbiota OR Gut Microbiota in Pregnancy OR Vaginal Microbiota OR Neonatal Immunity OR Immune Development in Infants OR Early-Life Immune Programming OR Allergy Risk OR Childhood Allergies OR Atopic Disease Development OR Breast Milk Microbiota OR Mode of Delivery OR Maternal Antibiotic Use OR Probiotic Supplementation in Pregnancy)
(6) Methods of review	
Details of methods, number of reviewers, how agreements are reached and disagreements are dealt with, etc.	Three main reviewers and a fourth to resolve any disagreements. Any outstanding disagreements on an article are resolved by the supervisor.
Quality assessment tools or checklists used with references or URLs	The protocol will define the method of literature critique/appraisal use, and the STROBE tool will be used for the relevant content and methodology used in the each of the papers to be reviewed.
Narrative synthesis details of what and how the synthesis will be performed	Narrative synthesis will be performed alongside any meta-analysis and will be carried out using a framework that consists of four elements. First, a theory is developed on how the intervention works, why, and for whom. Second, a preliminary synthesis of findings of the included studies is developed. Third, relationships within and between the studies are explored. Fourth, the robustness of the synthesis is assessed.
Meta-analysis details of what and how analysis and testing will be performed. If no meta-analysis is to be conducted, please give a reason.	Although a meta-analysis is planned, this will only become apparent when we see what data are extracted and made available from the systematic review. It is necessary to consider how heterogeneity will be explored.
Grading evidence system used, if any, such as GRADE	GRADE will be used for evidence assessment.
(7) Presentation of results	
Additional material summaries, tables, flowcharts, etc., to be included in the final paper	A flowchart of the whole process—protocol, data extraction, tables, and forest plots of studies—will be included in the final review.

## Appendix B

**Table A2:** PRISMA 2020 checklist.

Section and Topic	Item #	Checklist Item	Location Where Item Is Reported
TITLE			
Title	1	Identify the report as a systematic review.	Page 1; lines 2 to 3
ABSTRACT			
Abstract	2	See PRISMA 2020 for the abstract checklist.	Page 1; lines 18 to 34
INTRODUCTION			
Rationale	3	Describe the rationale for the review in the context of existing knowledge.	Page 1 and 2; lines 39 to 57
Objectives	4	Provide an explicit statement of the objective(s) or question(s) the review addresses.	Page 2; lines 57 to 63
METHODS			
Eligibility criteria	5	Specify the inclusion and exclusion criteria for the review and how studies were grouped for the syntheses.	Page 2 and 3; lines 85 to 100
Information sources	6	Specify all databases, registers, websites, organizations, reference lists, and other sources searched or consulted to identify studies. Specify the date when each source was last searched or consulted.	Page 2; lines 64 to 76
Search strategy	7	Present the full search strategies for all databases, registers, and websites, including any filters and limits used.	Page 2; lines 77 to 83
Selection process	8	Specify the methods used to decide whether a study met the inclusion criteria of the review, including how many reviewers screened each record and each report retrieved, whether they worked independently, and, if applicable, details of automation tools used in the process.	Page 3; lines 100 to 110
Data collection process	9	Specify the methods used to collect data from reports, including how many reviewers collected data from each report, whether they worked independently, any processes for obtaining or confirming data from study investigators, and, if applicable, details of the automation tools used in the process.	Page 3; lines 100 to 110
Data items	10a	List and define all outcomes for which data were sought. Specify whether all results that were compatible with each outcome domain in each study were sought (e.g., for all measures, time points, and analyses), and, if not, the methods used to decide which results to collect.	ND
	10b	List and define all other variables for which data were sought (e.g., participant and intervention characteristics, funding sources). Describe any assumptions made about any missing or unclear information.	ND
Study risk of bias assessment	11	Specify the methods used to assess the risk of bias in the included studies, including details of the tool(s) used, how many reviewers assessed each study, whether they worked independently, and, if applicable, details of automation tools used in the process.	Page 3; lines 113 to 123
Effect measures	12	Specify for each outcome the effect measure(s) (e.g., risk ratio, mean difference) used in the synthesis or presentation of the results.	ND
Synthesis methods	13a	Describe the processes used to decide which studies were eligible for each synthesis (e.g., tabulating the study intervention characteristics and comparing them against the planned groups for each synthesis (item #5)).	Page 3 and 4; lines 124 to 142
	13b	Describe any methods required to prepare the data for presentation or synthesis, such as the handling of missing summary statistics or data conversions.	Page 3 and 4; lines 124 to 142
	13c	Describe any methods used to tabulate or visually display results of individual studies and syntheses.	Page 3 and 4; lines 124 to 142
	13d	Describe any methods used to synthesize results and provide a rationale for the choice(s). If a meta-analysis was performed, describe the model(s), the method(s) to identify the presence and extent of statistical heterogeneity, and the software package(s) used.	Page 3 and 4; lines 124 to 142
	13e	Describe any methods used to explore possible causes of heterogeneity among study results (e.g., subgroup analysis, meta-regression).	ND
	13f	Describe any sensitivity analyses conducted to assess robustness of the synthesized results.	ND
Reporting bias assessment	14	Describe any methods used to assess the risk of bias due to missing results in a synthesis (arising from reporting biases).	Page 3; lines 114 to 123
Certainty assessment	15	Describe any methods used to assess certainty (or confidence) in the body of evidence for an outcome.	ND
RESULTS			
Study selection	16a	Describe the results of the search and selection process, from the number of records identified in the search to the number of studies included in the review, ideally using a flow diagram.	Page 4; lines 144 to 152
	16b	Cite studies that might appear to meet the inclusion criteria but were excluded, and explain why they were excluded.	Page 4; lines 144 to 152
Study characteristics	17	Cite each included study and present its characteristics.	Page 5 and 6; lines 182 to 186
Risk of bias in studies	18	Present assessments of the risk of bias for each included study.	Page 3; lines 113 to 123
Results of individual studies	19	For all outcomes, present for each study (a) the summary statistics for each group (where appropriate) and (b) an effect estimate and its precision (e.g., confidence/credible interval), ideally using structured tables or plots.	Page 5 to 7; lines 166 to 169
Results of syntheses	20a	For each synthesis, briefly summarize the characteristics and risk of bias among contributing studies.	Page 7; lines 188 to 231
	20b	Present results of all statistical syntheses conducted. If meta-analysis was performed, present each summary estimate and its precision (e.g., confidence/credible interval) and measures of statistical heterogeneity. If comparing groups, describe the direction of the effect.	ND
	20c	Present results of all investigations of possible causes of heterogeneity among study results.	ND
	20d	Present results of all sensitivity analyses conducted to assess the robustness of the synthesized results.	ND
Reporting biases	21	Present assessments of risk of bias due to missing results (arising from reporting biases) for each synthesis assessed.	ND
Certainty of evidence	22	Present assessments of certainty (or confidence) in the body of evidence for each outcome assessed.	ND
DISCUSSION			
Discussion	23a	Provide a general interpretation of the results in the context of other evidence.	Page 10; lines 238 to 247
	23b	Discuss any limitations of the evidence included in the review.	Page 10; lines 248 to 283
	23c	Discuss any limitations of the review processes used.	Page 11; lines 285 to 306
	23d	Discuss implications of the results for practice, policy, and future research.	Page 11; lines 307 to 321
OTHER INFORMATION			
Registration and protocol	24a	Provide registration information for the review, including register name and registration number, or state that the review was not registered.	Page 2; lines 64 to 67
	24b	Indicate where the review protocol can be accessed, or state that a protocol was not prepared.	Page 2; lines 64 to 67
	24c	Describe and explain any amendments to information provided at registration or in the protocol.	Page 2; lines 64 to 67
Support	25	Describe sources of financial or non-financial support for the review and the role of the funders or sponsors in the review.	Page 12; line 341
Competing interests	26	Declare any competing interests of review authors.	Page 12; line 351
Availability of data, code, and other materials	27	Report which of the following are publicly available and where they can be found; template data collection forms; data extracted from included studies; data used for all analyses; analytic code; any other materials used in the review.	ND

## Appendix C

**Table A3:** The Newcastle–Ottawa Scale for the included cohort of prospective and retrospective studies.

	Selection				Comparability	Outcome			Quality Score	Risk of Bias (0–3: High, 4–6: Moderate, 7–9: Low)
Article	Q1	Q2	Q3	Q4	Q5	Q6	Q7	Q8		
Nyangahu, 2019 [1]	(A) truly representative	(A) derived from the same population	*	*	One factor controlled (maternal diet)	*	Yes	*	Moderate Quality Study (6)	Moderate Risk
Edwards, 2017 [2]	(A) truly representative	(A) derived from the same population	**	**	Two factors controlled (maternal diet and antibiotic use)	**	Yes	**	Good Quality Study (8)	Low Risk
Jeong, 2021 [3]	(B) somewhat representative	(A) derived from the same population	*	*	One factor controlled (mode of delivery)	*	Yes	**	Good Quality Study (7)	Low Risk
Grech, 2021 [5]	(A) truly representative	(A) derived from the same population	**	**	Two factors controlled (breastfeeding and antibiotic exposure)	*	Yes	*	Good Quality Study (7)	Low Risk
DuPont, 2023 [6]	(B) somewhat representative	(B) from a different but comparable population	*	*	One factor controlled (maternal gut microbiota)	**	Yes	**	Moderate Quality Study (6)	Moderate Risk
Sinha, 2024 [17]	(A) truly representative	(A) derived from the same population	**	**	Two factors controlled (maternal antibiotic use and delivery mode)	**	Yes	**	Good Quality Study (8)	Low Risk
Aparicio, 2023 [19]	(A) truly representative	(A) derived from the same population	*	*	One factor controlled (maternal vitamin D levels)	**	Yes	*	Good Quality Study (7)	Low Risk
Halkjær, 2024 [26]	(B) somewhat representative	(B) from different but comparable population	*	*	One factor controlled	**	Yes	**	Moderate Quality Study (6)	Moderate Risk
Bertelsen, 2013 [56]	(A) truly representative	(A) derived from the same population	**	**	Two factors controlled (maternal probiotic supplementation and breastfeeding)	**	Yes	**	Good Quality Study (8)	Low Risk

Selection: Q1. Representativeness of the exposure cohort. Q2. Selection of the non-exposure cohort. Q3. Ascertainment of exposure. Q4. Demonstration that the outcome of interest was not present at the start of the study. Comparability: Q5. Comparability of the cohort based on the design or analysis. Outcome: Q6. Assessment of the outcome. Q7. Was the follow-up long enough for outcomes to occur? Q8. Adequacy of the follow-up of cohorts. * indicates that the corresponding quality component or criterion is partially met, and ** indicates that the corresponding quality component or criterion is fully met.

## Appendix D

**Table A4:** Bias of the included cross-sectional studies evaluated according to the Newcastle–Ottawa Scale.

	Selection				Comparability	Outcome		Quality Score	Risk of Bias (0–3: High, 4–6: Moderate, 7–9: Low)
	Q1	Q2	Q3	Q4	Q5	Q6	Q7		
Jeong, 2022 [3]	**	*	**	**	*	**	**	Good Quality Study (8)	Low Risk
Nunez, 2021 [4]	**	*	**	**	**	**	**	Good Quality Study (8)	Low Risk
Moher, 2009 [7]	*	*	**	*	*	**	**	Good Quality Study (8)	Low Risk
Brozek, 2020 [8]	**	**	**	*	**	**	**	Good Quality Study (8)	Low Risk
Stang, 2010 [9]	**	*	**	**	**	**	**	Good Quality Study (9)	Low Risk
Higgins, 2011 [10]	**	**	**	*	**	**	**	Good Quality Study (9)	Low Risk
Wilson, 2021 [11]	*	*	**	*	*	**	**	Moderate Quality Study (6)	Moderate Risk
Zhou, 2023 [12]	**	**	**	*	**	**	**	Good Quality Study (9)	Low Risk

Selection: Q1. Representativeness of the exposure cohort. Q2. Sample size. Q3. Ascertainment of exposure. Q4. Response rate. Q5. Ascertainment of the screening/surveillance tool. Comparability: Q5. The potential confounders were investigated by subgroup analysis or multivariable analysis. Outcome: Q6. Assessment of outcome. Q7. Statistical test. * indicates that the corresponding quality component or criterion is partially met, and ** indicates that the corresponding quality component or criterion is fully met.

## Appendix E

**Table A5:** The Cochrane Risk of Bias assessment tool for randomized trials (RoB 2).

Articles	Bias Arising from the Randomization Process	Bias Due to Deviations from Intended Interventions	Blinding of Participants and Personnel	Bias Due to Missing Outcome Data	Bias in Measurement of the Outcome	Bias in Selection of the Reported Result	Overall RoB
Jones, 2024 [13]	Low Risk	Low Risk	Low Risk	Low Risk	Low Risk	Low Risk	Low Risk
Embleton, 2023 [15]	Low Risk	Low Risk	Low Risk	Low Risk	Low Risk	Low Risk	Low Risk
Mueller, 2023 [16]	Low Risk	Low Risk	Low Risk	Low Risk	Low Risk	Low Risk	Low Risk
Mokkala, 2021 [20]	Low Risk	Low Risk	Low Risk	Low Risk	Low Risk	Low Risk	Low Risk
Sugino, 2022 [21]	Low Risk	Low Risk	Low Risk	Low Risk	Low Risk	Low Risk	Low Risk
Bisgaard, 2023 [41]	Low Risk	Low Risk	Low Risk	Low Risk	Low Risk	Low Risk	Low Risk
Wan, 2023 [43]	Low Risk	Low Risk	Low Risk	Low Risk	Low Risk	Low Risk	Low Risk
Sun, 2022 [44]	Low Risk	Low Risk	Low Risk	Low Risk	Low Risk	Low Risk	Low Risk

## Appendix F

**Table A6:** A full list of the studies contributing to the summary in Table 3.

Articles	Microbiota Factor	Outcomes Assessed	Effect Size
Wilson et al. (2021) [11]	Vaginal microbial transfer	Microbiome restoration in CS infants	RR 0.58
Zhou et al. (2023) [12]	Vaginal microbiota transfer	Neurodevelopment and microbiome	RR 0.67
Mueller et al. (2023) [16]	Vaginal seeding at birth	Microbial engraftment in neonates	OR 1.8
Liu et al. (2022) [29]	Vaginal seeding post-CS	Allergy and microbiota composition	OR 0.65
Carpén et al. (2022) [40]	Maternal intestinal flora transfer	Neonatal gut colonization	RR 2.84
Dierikx et al. (2021) [64]	Antibiotics timing during CS	Initial microbial colonization	OR 1.5
Fransson et al. (2022) [59]	CS-associated microbiota patterns	Microbiota variation and delivery	RR 5.3
Linnér et al. (2020) [30]	Skin-to-skin microbiota transfer	Preterm infant microbiota shifts	RR 0.13
Dominguez-Bello et al. (2020) [5]	Vertical transmission via skin contact	Maternal-offspring microbial overlap	OR 3.8
Hamidi et al. (2023) [31]	Antibiotic prophylaxis at CS	Gut microbiome composition in neonates	RR 0.72
Sinha et al. (2024) [17]	Microbiota reshaping in CS context	Immune and microbiota modulation	OR 4.1
Chen et al. (2024) [61]	Vaginal seeding for CS-born infants	Correction of abnormal microbiota	OR 0.3
Butler et al. (2020) [52]	Perception of vaginal seeding	Behavioral study on delivery options	OR 5.2
Butler et al. (2021) [51]	Diet and vaginal microbiota at delivery	Microbiota shift via delivery route	RR 2.94
Sun et al. (2022) [44]	Impact of birth mode on microbiome	Microbiome shaping by delivery mode	RR 0.70
Maqsood et al. (2022) [37]	Antibiotic use in mothers affects breast milk microbiota	Breast milk microbiome composition	RR 1.7
Gajecka et al. (2023) [27]	Maternal antibiotics and neonatal microbiome shifts	Neonatal microbiota composition	OR 2.1
DuPont et al. (2023) [6]	Antibiotic-induced dysbiosis and microbiome restoration	Reversal of maternal/infant dysbiosis	RR 4.10
Edwards et al. (2017) [2]	Antibiotic exposure influences maternal gut microbiota	Maternal gut microbiota variation	OR 0.9
Zijlmans et al. (2015) [62]	Perinatal antibiotic exposure and infant microbiota diversity	Reduced microbial diversity in neonates	RR 0.14
Embleton et al. (2023) [15]	Exclusive human milk diet in preterm infants	Gut microbiome composition in preterm infants	RR 3.25
Yeruva et al. (2023) [23]	Human milk miRNAs and microbiota modulation	Infant gut microbiota and growth	RR 1.7
Zamanillo et al. (2019) [58]	Breast milk microRNA influence on infant BMI and microbiota	Infant BMI and gut microbial changes	RR 2.3
Notarbartolo et al. (2022) [67]	Breast milk microbiota composition	Microbiota diversity and child health outcomes	OR 1.46
Davis et al. (2022) [69]	Breastfeeding effects on gut microbiome and immunity	Early immune development and gut colonization	OR 1.64
Sillner et al. (2021) [70]	Breastfeeding vs. formula feeding metabolite profiles	Microbial and metabolic profiles over time	OR 4.07
Ferretti et al. (2018) [66]	Mother-to-infant microbial transfer via breastfeeding	Infant gut colonization patterns	RR 2.78
Gilley et al. (2022) [14]	Breastfeeding and maternal obesity influencing microbiota	Infant gut microbiota variation in obese mothers	OR 2.92
Ji et al. (2023) [71]	Probiotics, prebiotics, and breastfeeding synergy	Health and gut microbiome improvements	RR 0.77
Bertelsen et al. (2013) [56]	Breastfeeding and probiotic milk reducing allergic diseases	Reduced allergic disease incidence in infants	RR 0.65
